# Bayesian Data Analysis for Revealing Causes of the Middle Pleistocene Transition

**DOI:** 10.1038/s41598-019-43867-3

**Published:** 2019-05-13

**Authors:** Dmitry Mukhin, Andrey Gavrilov, Evgeny Loskutov, Juergen Kurths, Alexander Feigin

**Affiliations:** 1 Institute of Applied Physics of the Russian Academy of Sciences, 603950 Nizhny Novgorod, Russia; 2 Potsdam Institute for Climate Impact Research, 14412 Potsdam, Germany

**Keywords:** Palaeoclimate, Nonlinear phenomena

## Abstract

Currently, causes of the middle Pleistocene transition (MPT) – the onset of large-amplitude glacial variability with 100 kyr time scale instead of regular 41 kyr cycles before – are a challenging puzzle in Paleoclimatology. Here we show how a Bayesian data analysis based on machine learning approaches can help to reveal the main mechanisms underlying the Pleistocene variability, which most likely explain proxy records and can be used for testing existing theories. We construct a Bayesian data-driven model from benthic *δ*^18^O records (LR04 stack) accounting for the main factors which may potentially impact climate of the Pleistocene: internal climate dynamics, gradual trends, variations of insolation, and millennial variability. In contrast to some theories, we uncover that under long-term trends in climate, the strong glacial cycles have appeared due to internal nonlinear oscillations induced by millennial noise. We find that while the orbital Milankovitch forcing does not matter for the MPT onset, the obliquity oscillation phase-locks the climate cycles through the meridional gradient of insolation.

## Introduction

**Figure 1:**
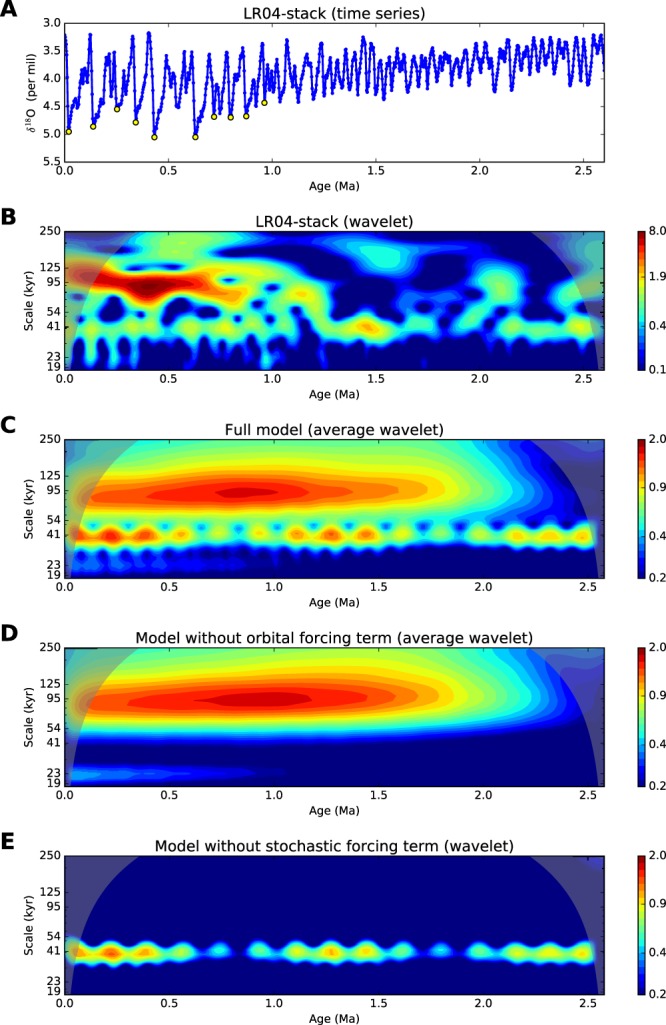
LR04 stack and output of the model. (**A**) LR04 time series of *δ*^18^O. The yellow circles mark the most rapid deglaciations with *δ*^18^O decrease faster than 1.1 per mil per 20 kyr. (**B**) Its wavelet power spectrum. (**C**) Model wavelet power spectrum averaged over 10000 model runs. (**D**) The same but with zero orbital forcing in the model. (**E**) The same but without the stochastic term in the model. Wavelet power is plotted in the logarithmic scale. Morlet wavelets were used via the Aaron O’Leary’s “Wavelets” Python module.

The pronounced change in the glacial-interglacial regime that occurred about 1 million years ago – the so-called Middle Pleistocene transition (MPT) – is widely regarded as an apparent manifestation of climate system’s nonlinearity. The MPT is observed in various proxy records as a shift in glaciation periodicity (from 41 kyr to approximately 100 kyr) accompanied by both an increase of the ice/temperature oscillation amplitude and a change of the characteristic shape of the oscillations from almost symmetrical to a saw-tooth shape with gradual coolings and rapid deglaciations (see Fig. 1(A,B) and refs^1–3^). The most significant external forcing of climate – the insolation variations affected by the Earth’s orbital parameters (the Milankovitch forcing) – remained unchanged during the Pleistocene. Hence, it is clear that the MPT is closely connected with the internal properties of climate and their possible response to large-scale changes of the environment. Currently, there are ongoing discussions concerning the mechanisms of the MPT and the roles of different orbital parameters and the natural climate variability in it. The problem is that the climate is a complex high-dimensional system with various nonlinear feedbacks; therefore, to identify the mechanisms one should distinguish the most important subsystems driving such changes. The latter is indeed a very problematic task due to difficulties with the verification of different models. Existing theories of the Pleistocene dynamics regard various internal factors for an explanation of MPT causes, such as the ice-albedo, precipitation-temperature and sea level feedbacks, atmospheric and ocean circulation, CO_2_ cycle, dust accumulation, etc. (cf. refs^2,4^). Many dynamical mechanisms of glacial cycles have been suggested based on different conceptual models derived from simplified physical considerations. In particular, they include relaxation oscillations arising under long-term trends in parameters (see the review^5^ of the corresponding models), nonlinear resonance to the orbital forcing^6^, noise- and forcing-induced transitions between multiple steady states^7^, chaotic response to the insolation forcing^8^, stochastic resonance^9^, etc. Several of the suggested models provide a good fit to the observed proxy records, but the ability of a model to reproduce data is certainly not sufficient for the verification of a theory. Tziperman *et al*.^10^ argue that the nonlinear phase-locking mechanism common for many nonlinear dynamical models can easily provide the correct output through synchronization of a model with the Milankovitch forcing, but the physical mechanism put in the model does not have to be necessarily correct.

To overcome this challenging puzzle, we here intend to explore the Pleistocene glacial cycles by Bayesian data analysis revealing the model that is minimal but sufficient for describing data. Mathematically, such a model provides the highest probability to produce the proxy records we have, and hence, yields statistically justified inferences. The advantages of the Bayesian methodology in selecting the proper scenario underlying the paleoclimate observations were discussed, e.g. in ref.^5^. Here we show how the data-driven model of the Pleistocene dynamics obtained from the Bayesian principles can be used for supporting or rejecting existing climatological theories.

We infer that nonlinear feedbacks in the climate system are principal factors for the MPT, whereas external forcing – the gradient of insolation – only paces the major deglaciations in the post-MPT climate. Thus, our objective analysis supports those theories bringing internal climate variability to the forefront, while those regarding the orbital oscillations as a main driver of the 100 kyr glacial-interglacial cycles are essentially rejected.

## Results

First, we describe the data we use and the dynamical model form we suggest. After that, we show how the model learned captures the main properties of the Pleistocene dynamics. Then we use the model for analyzing influences of different factors such as trends and forcings, as well as for studying the mechanism underlying the observed response. Finally, we present a prediction of climate cycles made by the model.

### Data and data-driven model

For the purpose stated above we restrict our consideration by the widely used LR04 stack of benthic *δ*^18^O records^11^. This stack accumulates data from 57 sites scattered over the globe and reflects the global average of total climate changes. We took only the last part of the time series beginning form 2.6 Ma when the glacial-interglacial cycles became regular^2^. Additionally, for technical reasons, we made this time series uniformly sampled with 2.5 kyr time step by means of applying the 5 kyr sliding window (see Methods). The latter smoothing does not disturb much the structure of the cycles, while just slightly decreases short-term noise in the data. The time series used for analysis together with its wavelet transform are shown in Fig. 1(A,B).

The data-driven model is constructed in the form of stochastic discrete dynamical system following the works refs^12–14^.

1 $${X}_{n}=f({X}_{n-1},\,\cdots \,{X}_{n-L},{t}_{n},{{\bf{q}}}_{n})+g({X}_{n-1},\,\cdots {X}_{n-L}){\zeta }_{n}\mathrm{.}$$

(i) The first term here is the deterministic evolution operator (dynamical system) mapping some history of climate’s states of duration *L* (the model dimension) to the next state. This term describes the internal dynamical properties of the system. The second term parameterizes the stochastic forcing of the model, which is needed to account for processes with time scales under the time resolution of our data, e.g. the millennial and centennial dynamics. Such a “noise” was shown to play a crucial role in the ice ages (see e.g. refs^5,7,9^), so the random perturbations are expected to be an essential part of the model. In the suggested model form such a noise can be state-dependent due to the product of the function *g* and uncorrelated Gaussian noise *ζ*. Both the functions *f* and *g* are unknown *a priori* and found by means of Bayesian machine learning techniques. In this work we define them via universal approximators (see Methods for details), that makes the form of the model Eq. 1 quite general and able to describe a wide class of dynamical systems.
(ii) Next, it was proposed by a number of theories of the MPT that the long-term Cenozoic cooling – e.g. a secular decrease in atmospheric pCO_2_^15^, global mean temperature^16^, deep water temperature^17^ – have brought the climate system to some critical transition after which the nonlinear oscillations of ice sheets became feasible. To reflect possible changes of this type, we make the deterministic part *f* of the model depend explicitly on time *t*_*n*_ by some modification of standard functions used for its approximation (see Methods). Such a time-dependence allows us to study a slow evolution of the climate system in time and thus to reveal dynamical mechanisms underlying the observed transitions. Moreover, it gives us an opportunity to extrapolate the model beyond the observations and predict the dynamical regime over some time interval in future^12–14^.
(iii) The last factor Eq. 1 depends on is an orbital forcing **q** – the Earth’s insolation variations. This signal is affected primarily by three astronomical parameters: precession of both the Earth’s axis and orbit yielding together 19 and 23 kyr spectral peaks, obliquity oscillations with the 41 kyr dominating period, and less-powerful variations of the Earth’s orbit eccentricity contributing to time scales around 100 kyrs (see power spectra of the insolation in Fig. S3). Although the insolation signals have the same spectral components over the globe, the relationships between different harmonics are latitude-dependent: in particular, the obliquity peaks being strong far from the equator vanish in the tropics. We take into account such a dependence by using a two-dimensional forcing **q** taken from the dataset described in ref.^18^ consisting of July insolation time series at the tropical (15°N) and sub-polar (65°N) latitudes.

The complexity of the model Eq. 1 is determined by a set of the structural parameters – the dimension *L* and the numbers of nonlinear elements in the approximators of both functions *f* and *g* parameterizing the deterministic and stochastic parts respectively (see Methods and Supplementary Information (SI)). For the selection of the optimal model’s structure, we use Bayesian methodology: the optimal model is the one that maximizes the probability of generating the time series in hand; see the section Methods for details and practical implementation of the approach.

Regarding the LR04 dataset analysis presented here, the model Eq. 1 was trained with different sets of structural parameters. Then the different models were compared in accordance with the Bayesian criterion (see methods and SI) and the top-10 best models were considered further. All the models considered demonstrate qualitatively very similar results as well as the same mechanism of the MPT; therefore, hereinafter we illustrate the results using the best model only.

### Dynamics of the model

**Figure 2:**
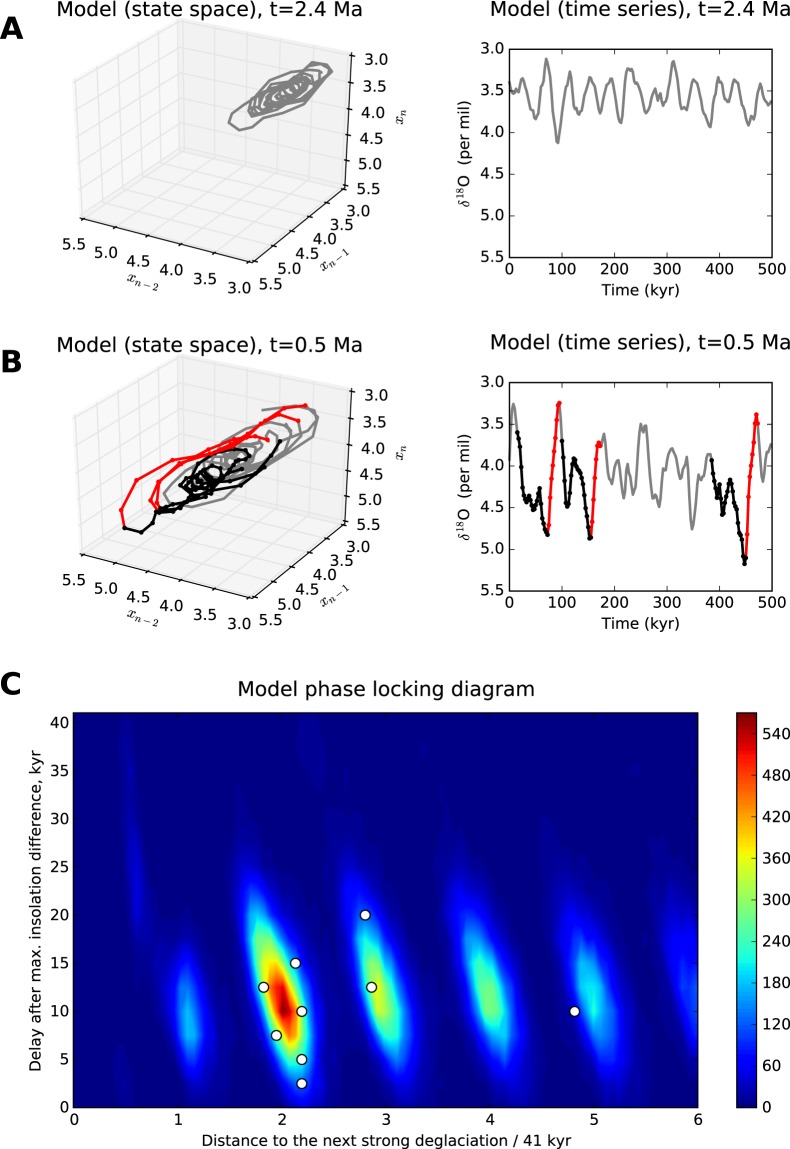
Model’s glacial-interglacial cycles. (**A**) 500 kyr fragment of a model trajectory for the early Pleistocene (2.4 Ma): a three-dimensional projection of the model’s phase space (left) and corresponding time series (right). (**B**) The same but for the late Pleistocene (0.5 Ma). Gradual glaciations (increases of *δ*^18^O) and rapid deglaciations are highlighted in black and red respectively. (**C**) Statistics of the major deglaciations defined as in Fig. 1(A): joint distribution density of deglaciation delay after the closest insolation gradient maximum and the time distance to the next deglaciation (measured in 41 kyr periods). The corresponding major deglaciations in the LR04 stack (see the yellow circles in Fig. 1(A)) are shown by circles.

The model Eq. 1 is the stochastic dynamical system, hence its output depends on the random variable *ζ* and can differ from run to run. Figure 1(C) shows the wavelet transform (WT) of the model averaged over an ensemble of 10,000 model runs with different noise realizations, as compared to the WT of the single LR04 data (Fig. 1(B)). Due to averaging effects, WT of the model looks more “homogeneous” than that of the data (and that of individual model time series; see an example in Fig. S1) and allows a clear identification of the MPT onset in the model behavior. It is seen that the model reproduce the 41 kyr spectral line during the entire Pleistocene associated with the linear response to the obliquity signal as well as the moving of the substantial part of the spectral power to lower frequencies that culminates in the interval from 1.3 to 1 Ma – the main manifestation of the MPT. Beyond that, the model reproduces correctly the changing characteristic shape of the climate cycles, as shown in Fig. 2(A,B): close to sinusoidal symmetric oscillations during the pre-MPT epoch and sawtooth long-period and large-amplitude motions with the rapid deglaciations (*δ*^18^O decreases) after the MPT.

### The impact of stochastic forcing

To study the role of the random fluctuations (noise) in the model, which represent the sub-grid millennial and centennial climate variability, we compare outputs of the model in three variants: (i) the full model containing all the forcings, (ii) the model without the insolation forcing, in which both forcing time series entering **q** are set to their mean values, and (iii) the noise-free model with *g* = 0. We find that the stochastic forcing is a crucial factor for the frequency-band change associated with the MPT: the transition to the 100 kyr scale occurs in the same way in the model without any insolation signal, whereas the model with insolation forcing only and no noise only exhibits a response to the obliquity 41 kyr oscillations through the entire Pleistocene (Fig. 1(D,E)). Thus, the model internal dynamical properties, short-scale climate fluctuations and the model nonstationarity induce together the frequency shift in the *δ*^18^O variations; by contrast, the variability in insolation is not instrumental in the MPT.

**Figure 3:**
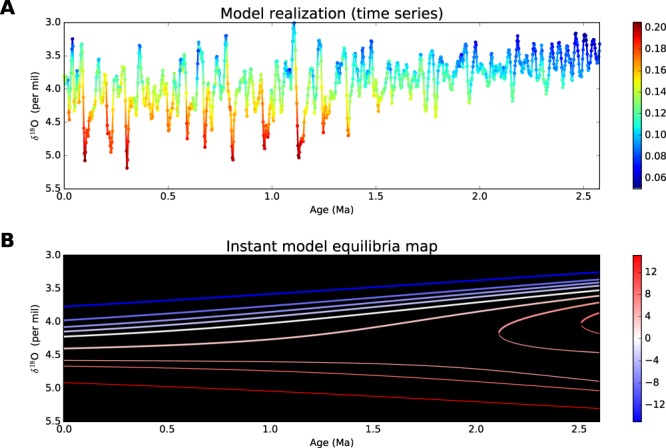
Deterministic and stochastic model specifics. (**A**) An example of model time series (the same time series is used in Fig. S1) colored in accordance with the noise power: color represents the values of the “instantaneous” noise variance *g*^2^ in Eq. 1 normalized to the variance of the LR04 stack. (**B**) Deterministic model’s (see the text) steady states at different insolation gradient forcing levels (colors correspond to the insolation gradient values). The stable and unstable steady states are shown by thick and thin lines respectively.

The resulting probability distribution of noise in the model Eq. 1 is not constant in time due to the state-dependence of the noise amplitude *g*. The ratio of the “instantaneous” noise variance *g*^2^ to the variance of the LR04 time series is marked by color in Fig. 3(A), where the time series from a single randomly chosen model run is plotted. The increase in the millennial variability in colder climate seen in Fig. 3(A) coincides well with findings of refs^19,20^ showing an amplification of the millennial-scale climate variability amplitude with an increasing of the continental ice mass. As shown below, such an increase in the sub-grid fluctuations may contribute to but does not play a central role in the MPT mechanism.

### The impact of insolation forcing

Although the insolation signals **q** at the input of the model Eq. 1 are comprised of two distinct time series, we found that the optimal models only depend on a particular combination of these factors that is close to the difference between the tropical and sub-polar insolation inputs (see SI). Thus, in accordance with our analysis, the only insolation forcing that matters in the Pleistocene climate is the meridional gradient of the insolation mainly affected by the obliquity oscillations (see Fig. S3). All the harmonics related to the precession and eccentricity forcings nearly disappeared in the gradient and thereby cannot contribute to the observed dynamics. The insolation gradient was regarded as a main driver for the 41 kyr climate cycles in the pre-MPT epoch (from 3 Ma to 0.8 Ma), through its modulation of the atmospheric meridional heat and moisture fluxes^21^. Our results indeed provide further direct empirical evidence from the LR04 stack that such a forcing remains dominant through the rest of the Pleistocene too.

In spite of the fact that the insolation forcing is not responsible for the MPT onset, it contributes significantly to post-MPT most powerful glacial-interglacial cycles via the phase-locking mechanism through which the origins of the major deglaciations follow the maxima of the insolation gradient time series. To illustrate this, we considered an ensemble of major deglaciations with 1 per mil *δ*^18^O decrease per 20 kyr or faster in the model behavior, collected from 10,000 model time series over the considered time interval from 2.6 Ma to present. For each event from this ensemble we calculated the time to the closest insolation gradient maximum as well as the time to the next deglaciation from the ensemble. A joint distribution density of these values shown in Fig. 2(C) indicates that the major deglaciations occur, on average, 10 kyr after the insolation gradient maxima and, consequently, the periods of glacial-interglacial cycles are close to multiples of the obliquity period 41 kyr. Moreover, most of the periods are distributed around the doubled and tripled obliquity period; this results in the near 100 kyr line in the mean model wavelet spectrum (Fig. 1(C)). The corresponding 10 major deglaciations from the LR04 stack are marked by circles in both *δ*^18^O time series (Fig. 1(A)) and the density plot in Fig. 2(C): they fall well within the model statistics of deglaciation times.

### Dynamical mechanism of the MPT

To identify a mechanism underlying the model’s behavior described above, let us first look at the steady states of the deterministic part of the model *X*_*n*_ = *f*(*X*_*n*−1_, … *X*_*n*−*L*_, *t*_*n*_, **q**_*n*_) at different constant values of the insolation forcing **q**. Figure 3(B) shows the time evolution of the steady states at different insolation gradient levels, representing a “skeleton” of the obtained model’s dynamics. While at the low insolation gradient values, corresponding to warmer climate, the model is stable, the stability of the steady state decreases with the insolation gradient, and eventually, it becomes unstable at high insolation gradient levels. Nevertheless, the deterministic model under the quasi-periodical insolation gradient always lives near the “warm” stable state: the relatively short period of the forcing prevents it from going far from it in spite of the epochs of the model’s instability. Instead, the model state just slightly moves along with the insolation gradient oscillations. This is why the model demonstrates the 41 kyr response plotted in Figs 1(E) and 2(A) during the whole Pleistocene in the absence of the stochastic part.

The behavior changes essentially if the millennial processes are switched on. Due to the trend in the model, the steady states corresponding to stable and unstable epochs of climate converge with time (as seen e.g. from Fig. 3(B)); simultaneously, the stability of the warm climate states decreases and the amplitude of the millennial noise increases (Fig. 3(A)). Eventually, starting from the middle Pleistocene, the noise is able to push out the model farther from the steady state to the area where the response of the model is essentially nonlinear: fast relaxations from the cold to warm climate and slower backward motions occur. The result is that in the full model with both millennial and insolation forcings we see the onset of the regime with noise-induced sawtooth oscillations (Fig. 2(B)) associated with the MPT.

In general, the insolation forcing is not necessary for the MPT: the decreased stability of the warm steady state allows the noise to provide the transition starting from the average insolation gradient level (see e.g. Fig. 1(D)). However, the insolation gradient paces the glacial-interglacial cycles in the following way. The fastest stages of the glacial-interglacial oscillations (i.e., the rapid deglaciations) tend to occur when the climate is cold enough (high *δ*^18^O level) and, at the same time, the insolation gradient has passed the maximal phase approaching the average. Under such conditions, the glacial climate is forced to go rapidly toward the warm steady state which appears in the model at the average insolation gradient level. Both these conditions determine the phase locking of the major deglaciations and the obliquity signal, which we observe in the model dynamics as well as in the LR04 time series (Fig. 2(C)). This mechanism is further explained in SI (the text and Figs S4–S6).

### Model prediction

Finally, let us look what the obtained model predicts, being simply extrapolated into future, under the assumption that the detected trend stays the same. Note that this assumption may not be realistic, since there is no clear evidence of the ongoing long-term cooling even in the late Pleistocene^1^. It is even more suspect today, when the anthropogenic warming contributes to shifting the climatic means. Still, if such a trend held steady in future, the large glaciations in the model would still occur, since warm and glacial states would continue to converge as Fig. S7 indicates. While the amplitude of “future” climate variability is almost unchanged in time, an additional short-scale (around 20 kyr period) cycle appears in the model time series independently of the insolation forcing (see WT for the model extrapolations with and without the insolation forcing in Fig. S7).

## Discussion

The optimal Bayesian data-driven model derived here from the LR04 stack shows that strong nonlinear feedbacks in climate, gradual trends of global cooling, stochastic and insolation (obliquity) forcings are all important for various aspects of the MPT. From a dynamical point of view, the MPT is shown to be generated due to a long-term trend in climate leading to a noise-induced nonlinear oscillation build-up. This trend makes the warm steady states less stable and hence allows the climate to reach colder states. As a result, the slow onset/rapid termination glacial-interglacial climate oscillations of large amplitude became more approachable in the late Pleistocene; this gives indication that the large glaciations started to be triggered at higher temperatures than in the pre-MPT epoch. This conclusion supports indirectly the hypothesis put forth in ref.^17^ about the leading role of a gradual deep ocean cooling in the MPT, through decreasing the ocean heat capacity and hence allowing sea ice to grow at higher atmospheric temperatures.

However, the strongly nonlinear relaxation oscillations responsible for the large sawtooth glacial-interglacial cycles cannot arise in our model without energetic millennial (and/or centennial) climate variability, represented by a stochastic process. So, the data-driven modeling of such short-scale variability (e.g. Dansgaard-Oeschger, Heinrich, Bond oscillations), with connection to large glacial-interglacial cycle models, could be helpful for further clarification of the mechanisms underlying the Pleistocene dynamics.

Regarding the insolation forcing, we have found that only the insolation gradient, driven primary by the obliquity oscillations, is important for the Pleistocene dynamics. For the pre-MPT 41 kyr world the principal role of insolation gradient was explained in ref.^21^, but the physical mechanism for its dominance in the late Pleistocene (shown by our model) is not presently clear. Accordingly to ref.^22^, the glacial oscillation is phase locked with the Milankovitch forcing via the high-latitude insolation: lower insolation leads to larger glacier growth and makes the sea ice switches (rapid expansions of sea ice) followed by the deglaciation stages more probable than at higher insolation values. But the high-latitude insolation signal is dominated by the precession rather than obliquity oscillation, whereas our analysis uncovers that the impacts of both precession and eccentricity forcings are negligible. To reconcile these differences, we conjecture here that the tropical insolation can be also important in this mechanism. Above the average tropical insolation in combination with the low high-latitude insolation may result in an intensification of moisture transport to high latitudes and a related increase in the ice accumulation rate. The latter prolongs the stability of continental ice allowing temperatures to reach much lower values through the ice-albedo feedback. Eventually, all behavior explained in refs^4,17,22^ still happens – the low temperatures, large sea ice extent, reducing precipitations followed by retreating of the glaciers, but the deglaciation stage starts from much colder conditions yielding higher amplitudes of the glacial cycles. Thus, the major deglaciations are tied to the positive phase of the low-high latitude insolation difference (i.e. the insolation gradient) oscillation, providing a phase locking of the climate cycles with the obliquity cycles. Probably, this is the direction the conceptual models could be modified in.

All the conclusions we made in this work are inferred by the analysis of the LR04 stack which only represents the global climate changes during the Pleistocene. Therefore the dynamical model derived from this data set can only describe the salient properties of the climate system and necessarily lacks potentially important regional details. In particular, it was shown^3^ that the Ocean Drilling Program records in the Southwest Pacific Ocean – an important region for studying the global ocean circulation – indicate a much more abrupt change in *δ*^18^O from 950 ka to 870 ka compared to much smoother globally-averaged changes. No doubt, differences in regional climate variability can be important for more detailed studies of glacial mechanisms, and future works on data-driven modeling of paleoclimate would be focused on the analysis of multivariate time series over the globe.

Another question beyond the scope of this work concerns the reversibility of the MPT: whether is it possible to bring the climate back to the 41-kyr world by reversing the long-term trend in the system? In principle, the dynamics of the model obtained here is reversible in this sense: if we initialize the model by current conditions and launch the sequence {*t*_*n*_} in Eq. 1 backward, we get the completely reversed scenario of the MPT, as shown in Fig. S8. This means that structurally, there is no hysteresis, or multistability, in the model on the time interval corresponding to the MPT. But the problem is that the LR04 time series does not provide sufficient information for identification of the physical processes contributing to the trend itself, whereas some of them may be irreversible, e.g. glacial erosion of regolith which has been suggested as a primary factor for large glaciations^1^. Further understanding and modeling the causes of such long-term changes of the system may shed new light on this task.

## Methods

In this section we first describe a specific form of the data-driven model we use. Then we briefly explain the representation of model structural parts, an algorithm for model learning and optimization, as well as a data preparation procedure. Details of the methods for representation, learning and optimization of the model in the form of Eq. 1 can be found in refs^12–14,23,24^.

### Model structure

The stochastic evolution operator (EO) we reconstruct from data (Eq. 1) explicitly depends on time as well as the forcing **q**. The dependence on time is needed for parameterizing possible deformations of the EO due to slow trends in climatic conditions. A response of the system to such trends can be essentially state-dependent. Hence, the time variable *t* should be passed to the input of the model together with the state variables *X*. Obviously, the response of the system to forcing (insolation signal in the present case) can be state-dependent as well, and the forcing **q** should be also involved as a dynamic variable. However, we should exclude here odd models which allow the astronomically driven forcing *q* to respond to the climatic trends or, vice versa, permit a direct impact of the stationary forcing on much longer-scale trends. To this end, we split the deterministic part *f* of the model into two terms, each of them being responsible for either trend or insolation forcing impact:

2 $$f({X}_{n-1},\,\cdots {X}_{n-L},{t}_{n},{{\bf{q}}}_{n})={f}_{1}({X}_{n-1},\,\cdots {X}_{n-L},{t}_{n})+{f}_{2}({X}_{n-1},\,\cdots {X}_{n-L},{{\bf{q}}}_{n})\mathrm{.}$$

### Model representation

For the parameterization of *a priori* unknown functions *f*_1_, *f*_2_ and *g* in Eqs 1 and 2 we use a simple artificial neural network (ANN) in the form of perceptron with one hidden layer and hyperbolic tangent activation function, which is known to be a universal approximator^25^:

3 $$\phi (z)=\sum _{i\mathrm{=1}}^{m}\,{\alpha }_{i}\cdot \,\tanh ({\omega }_{i}^{T}z+{\gamma }_{i}\mathrm{).}$$

Here *m* is a number of neurons, (*α*, *ω*, *γ*) are the fitted coefficients (model’s parameters), and *z* is an input of some dimension *d*. Since we analyze the single LR04 time series, hereinafter we use the scalar variant of the function *φ*: **R**^*d*^ → **R**, so that *α* ∈ ***R***, *ω* ∈ **R**^*d*^, *γ* ∈ **R**. While the functions *f*_1_ and *g* take the vector of Takens variables^26^ *z* = (*X*_*n*−1_, … *X*_*n*−*L*_) as an input (i.e. *d* = *L*), the combined vector *z* = (*X*_*n*−1_, … *X*_*n*−*L*_, **q**_*n*_) is passed to the input of the function *f*_2_, so *d* = *L* + 2 for *f*_2_ due to two-dimensional forcing **q** (see the main text).

The explicit dependence on time *t* of the deterministic model (Eq. 2) is put in *f*_1_ by means of a modified ANN structure (Eq. 3) as follows:

4 $$\phi (z,t)=\sum _{i\mathrm{=1}}^{m}\,({\alpha }_{i}^{1}+{\alpha }_{i}^{2}\cdot t)\cdot \,\tanh ({\omega }_{i}^{T}z+{\gamma }_{i}\mathrm{).}$$

In fact, this is the first-order (linear) expansion of a weak time-dependence of the model caused by slow trends in the system. It was shown in refs^27,28^ that such an approximation is efficient for modeling and prediction of low-dimensional nonlinear dynamical system of general type with slowly changing parameters.

### Model learning and optimization

Let us denote the model parameters entering in the functions *f*_1_, *f*_2_ and *g* by ${\mu }_{{f}_{1}}$, ${\mu }_{{f}_{2}}$ and *μ*_*g*_ respectively (each of these *μ* consists of corresponding ANN coefficients). To determine the parameters, we use a cost-function in a form of the Bayesian posterior probability density function (PDF):

5 $$P({\mu }_{{f}_{1}},{\mu }_{{f}_{2}},{\mu }_{g}|X,{\bf{q}})\propto P(X|{\mu }_{{f}_{1}},{\mu }_{{f}_{2}},{\mu }_{g},{\bf{q}})\times {P}_{pr}({\mu }_{{f}_{1}},{\mu }_{{f}_{2}},{\mu }_{g}\mathrm{).}$$

The first term in the right-hand side is the likelihood function – the PDF of obtaining data *X* = (*X*_1_, … *X*_*N*_) of duration length *N* by the model with parameters $({\mu }_{{f}_{1}},{\mu }_{{f}_{2}},{\mu }_{g})$ given the forcing time series **q** = (**q**_1_, … **q**_*N*_). This function can be inferred directly from Eqs 1 and 2 on the assumption that the random process *ζ* in Eq. 1 is Gaussian and uncorrelated:

6 $$\begin{array}{l}P(X|{\mu }_{{f}_{1}},{\mu }_{{f}_{2}},{\mu }_{g},{\bf{q}})=C{[\prod _{n=L+1}^{N}2\pi g{({x}_{n-1},\ldots {x}_{n-L},{\mu }_{g})}^{2}]}^{-\mathrm{1/2}}\\ \times \,\exp \{-\frac{1}{2}\sum _{n=L+1}^{N}\frac{{({x}_{n}-{f}_{1}({x}_{n-1},\ldots {x}_{n-L},{t}_{n},{\mu }_{{f}_{1}})-{f}_{2}({x}_{n-1},\ldots {x}_{n-L},{{\bf{q}}}_{n},{\mu }_{{f}_{2}}))}^{2}}{g{({x}_{n-1},\ldots {x}_{n-L},{\mu }_{g})}^{2}}\},\end{array}$$

where *C* is a constant depending on a starting fragment *X*_1_, …*X*_*L*_ of the time series *X* (see refs^23,24^). The second term in Eq. 5 is the prior PDF of the model parameters. This function restricts the domain of model learning in the space of parameters, thus compensating the degeneration of the ANN’s parameter space and simplifying the numerical analysis. Following refs^12–14,23,24,27^, we use a Gaussian prior PDF with different variances for different groups of ANN coefficients (*α*, *ω*, and *γ*).

A crucial point in data-driven modeling is selecting the model structure of optimal complexity in the sense of its correspondence to the data. The Bayesian way for optimal model selection is finding the model that maximizes the marginal likelihood function characterizing the probability to produce the data *X* by the model:

7 $$P(X|{{\bf{H}}}_{i})=\int \,P(X|\mu ,{{\bf{H}}}_{i})P(\mu |{{\bf{H}}}_{i})d\mu \mathop{\to }\limits^{{{\bf{H}}}_{i}}max\mathrm{.}$$

Here *μ* are the internal parameters of a particular model **H**_*i*_, *P*(*μ*|**H**_*i*_) is a prior probability density for them, and *P*(*X*|*μ*, **H**_*i*_) is the likelihood for the i_*th*_ model’s parameters. The predefined set of models {**H**_*i*_} should be wide enough to incorporate as much as possible physically relevant evolution operators of the system. Using condition Eq. 7 as a criterion for best model selection prevents us from obtaining overfitted models that fit well the particular observations (and hence yield large values of the likelihood in Eq. 7) but are useless for inferring robust dynamical laws underlying data.

The structural parameters determining our model complexity are the time lag *L* defining the model memory and the numbers of neurons ${m}_{{f}_{1}}$, ${m}_{{f}_{2}}$ and *m*_*g*_ in the ANNs representing both the deterministic (*f*_1_ and *f*_2_) and stochastic (*g*) parts of the model. Thus an ensemble of models used for the best model selection consists of the models with different structural parameter sets $(L,{m}_{{f}_{1}},{m}_{{f}_{2}},{m}_{g})$. The procedure for the estimation of the integral in Eq. 7 we use here is the same as in refs^23,24^ for every model **H**_*i*_ from an ensemble of $(L,{m}_{{f}_{1}},{m}_{{f}_{2}},{m}_{g})$ the following function derived by the Laplace integration method is calculated:

8 $$\begin{array}{rcl}-\,\mathrm{ln}\,P(X|{{\bf{H}}}_{i}) & \cong  & {\rm{\Psi }}({\mu }_{0})+\frac{1}{2}\,\mathrm{ln}\,|\frac{1}{2\pi }\nabla {\nabla }^{T}{\rm{\Psi }}({\mu }_{0})|,\\ {\rm{\Psi }}(\mu ) & = & -\mathrm{ln}[P(\mu |X,{\bf{q}},{{\bf{H}}}_{i})],\\ \mu  & = & ({\mu }_{{f}_{1}},{\mu }_{{f}_{2}},{\mu }_{g}\mathrm{).}\end{array}$$

Here *μ*_0_ is model parameters minimizing the function Ψ(*μ*), i.e. the cost-function Eq. 5, ∇∇^*T*^Ψ is the matrix of the second derivatives (Hessian matrix) of the function Ψ with respect to the parameters *μ* at the point of its minimum *μ*_0_. While the value of the first term in the upper row of Eq. 8 indicates how well the model outputs fit to the data, the second term penalizes the model for its complexity. Actually, the minimization of -ln *P*(*X*|**H**_*i*_) provides a balance between the fit accuracy and the model complexity.

Eventually, to obtain the best model, we have to learn each model from the ensemble, which gives us the values of *μ*_0_, calculate the optimality (Eq. 8), and select the model that minimizes the optimality.

### Data preparation

The original LR04 stack is sampled with non-constant time step ranging from 1 kyr to 2.5 kyr for the time period [2.6 Ma, 0 Ma]. Each point of these series represents the average value of *δ*^18^O on the corresponding time interval. Thus, we can consider the stack as a set of such time intervals. In order to construct the model described above, we resampled the LR04 stack with the constant time step 2.5 kyr and applied a smoothing window with the size *w* = 5 kyr simultaneously by the following procedure. For each time instance *t* of the new time series we took all time intervals of the original LR04 stack which intersect with or lie in [*t* − *w*/2, *t* + *w*/2]; the values from these intervals were averaged with the weights proportional to the sizes of the corresponding intersections and the resulting average value was taken as the new value at the time *t*.

The insolation forcing time series was resampled from the time step 1 kyr to the time step 2.5 kyr using the classic cubic spline interpolation.

## Supplementary information

Supplementary Information

## Data Availability

The analyzed LR04 stack is open to the public.
