# Supplementary material for: Bayesian Data Analysis for Revealing Causes of the Middle Pleistocene Transition
Source: Sci Rep. 2019 May 13;9:7328. doi: 10.1038/s41598-019-43867-3 (PMC6513842; doi:10.1038/s41598-019-43867-3)
Supplement: Supplementary file 1 — Supplementary Information [file 41598_2019_43867_MOESM1_ESM.pdf]

Supplementary Information

Bayesian Data Analysis for Revealing Causes of the Middle Pleistocene Transition

Dmitry Mukhin[1,*], **Andrey Gavrilov**[1], **Evgeny Loskutov**[1], **Juergen Kurths**[2,1], **and Alexander Feigin**[1]

[1]Institute of Applied Physics of the Russian Academy of Sciences, 603950 Nizhny Novgorod, Russia
[2]Potsdam Institute for Climate Impact Research, 14412 Potsdam, Germany
[*]mukhin@ipfran.ru

Supplementary text

Resulting model parameters. The optimal solution was achieved by performing totally 48,600 learning iterations covering the range of structural parameters $L = 1 \div 15$, $m_g = 0 \div 4$, $m_{f_1} = 1 \div 5$, $m_{f_2} = 1 \div 5$. Fig. S2 displays the dependence of the optimality cost function Eq. 8 on the structural parameters for the ensemble members in the vicinity of the optimum: the 10 most optimal solutions correspond to $L = 10 \div 11$, $m_g = 1$, $m_{f_1} = 1 \div 2$, $m_{f_2} = 1 \div 3$. The best model (the source of figures in this work) has $L = 10$, $m_g = 1$, $m_{f_1} = 1$, $m_{f_2} = 2$.

Resulting insolation forcing. We have discovered that in the top-10 optimal models the EO is depending mainly on the IG. In some of these models it can be inferred directly from the obtained functional form (the weights ω at the $\mathbf{q}$-variable), for the other models it can also be checked by computing the 2-D derivative $\nabla_{\mathbf{q}} f(X_{n-1}, \cdots X_{n-L}, t_n, \mathbf{q}_n)$ and comparing its direction with the direction of insolation gradient$q_2 - q_1$ (for example, by estimating the plane angle between them). For each of the top-10 optimal models we performed this computation by taking the ensemble of the observed data points (LR04 stack and the insolation forcing) as an input, obtained the ensemble of angles and calculated its mean value and standard deviation. In each case the absolute value of the mean angle between the derivative and the insolation gradient did not exceed 1.2^o and its deviation was no more than 2.75^o, i.e the angle is much less than 90°. This result allows us to conclude that the dependence of EO on the insolation forcing is mainly the dependence on the IG.

Explanation of the MPT dynamical mechanism. It is shown in Fig. 3 (B) that the deterministic model's

$$X_n = f(X_{n-1}, \cdots X_{n-L}, t_n, \mathbf{q}_n)$$

steady states at different constant values of the insolation gradient become closer to each other (converge) with time, so the phase space of the model "shrinks". While at the low insolation gradient the model's steady state is just a stable focus corresponding to warm climate, it looses stability with increase of the insolation gradient through the Neimark-Sacker bifurcation.[1] As a result, at the insolation gradient fixed in its maximal value the deterministic model has an unstable focus and an attractor in a form of the limit cycle (see Figs. S4 (B-D) and S5). But the deterministic model with the quasi-periodical insolation gradient forcing never approaches such an attractor: the relatively short period of the forcing prevents the phase trajectory from going far from the "warm" steady state. Instead, the model state lives on a slow manifold around it just slightly moving towards instantaneous attractor that changes with the forcing (Fig. S4 (A)). This leads to the 41 kyr response of the deterministic model (see Fig. 1 (E) and Fig. 2 (A)).

The slow trend in the obtained deterministic model decreases stability of the low insolation gradient steady states with time, so that the noise in the model Eq. 1 enables the phase trajectory to discover larger area around the steady state (Fig. S6). Due to nonlinearity of the model, this area of the phase space is not uniform: there are fast relaxation motions from the cold states (see red lines in Fig. S4 (B-C)) and slower backward motions. Eventually, starting from the middle Pleistocene the noise is able to push out the model to the motions of this kind even at average insolation gradient levels, as shown in Fig. S6, and so, the model demonstrates the noise-induced sawtooth relaxation oscillations plotted in Fig. 2 (B) and Fig. S6 (A). The insolation gradient forcing amplifies this effect: if climate becomes cold enough at the highest IG, then it relaxes more rapidly to the warm stable steady state arisen near the average IG. This leads to the phase locking of such major deglaciations and the insolation gradient signal (see the main text of the article).

References

1. Kuznetsov, Y. A. & Sacker, R. J. Neimark-Sacker bifurcation. *Scholarpedia* **3**, 1845 (2008). Revision #91556.

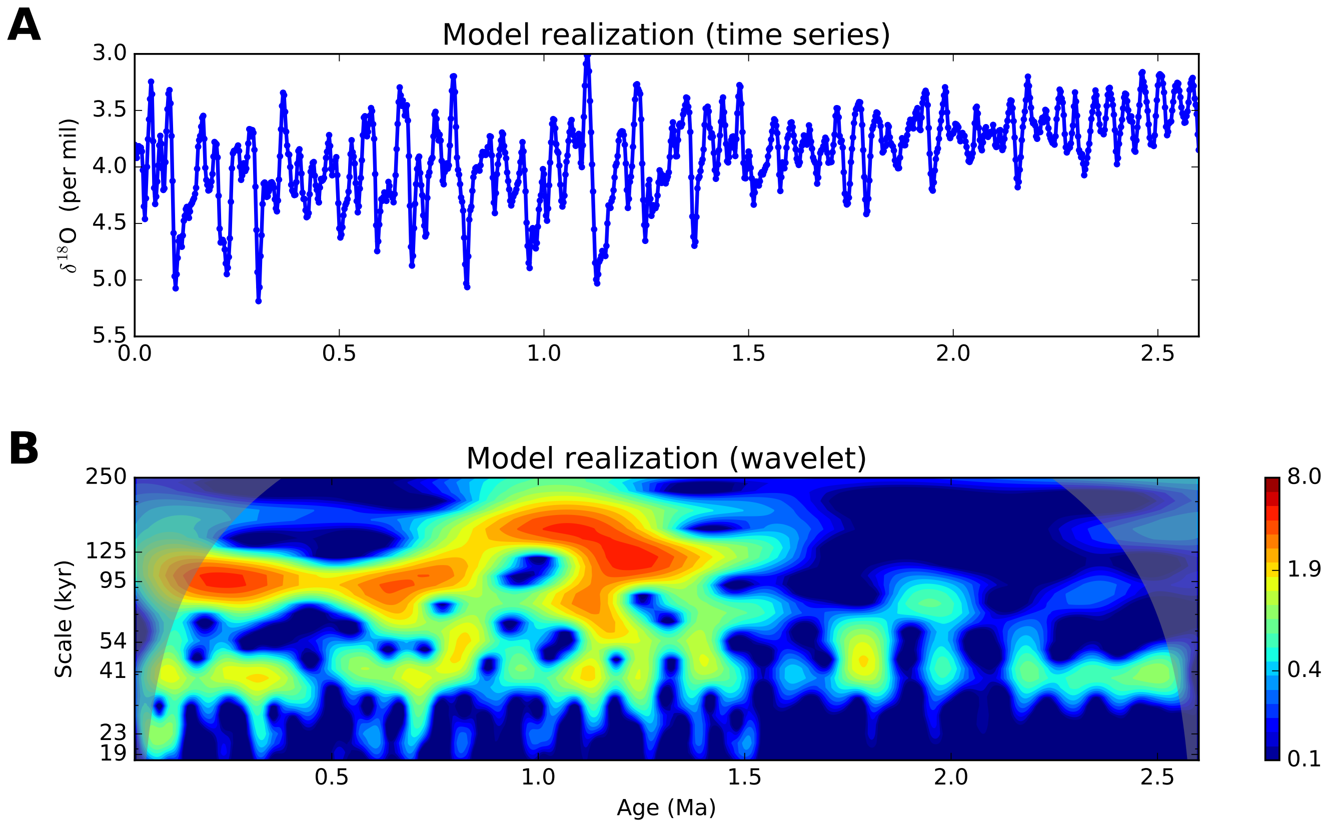

Figure S1. (**A**) An example of a particular model Eq. 1 time series from the ensemble used in Fig. 1 (C) and Fig. 2 (C). (**B**) Its wavelet transform.

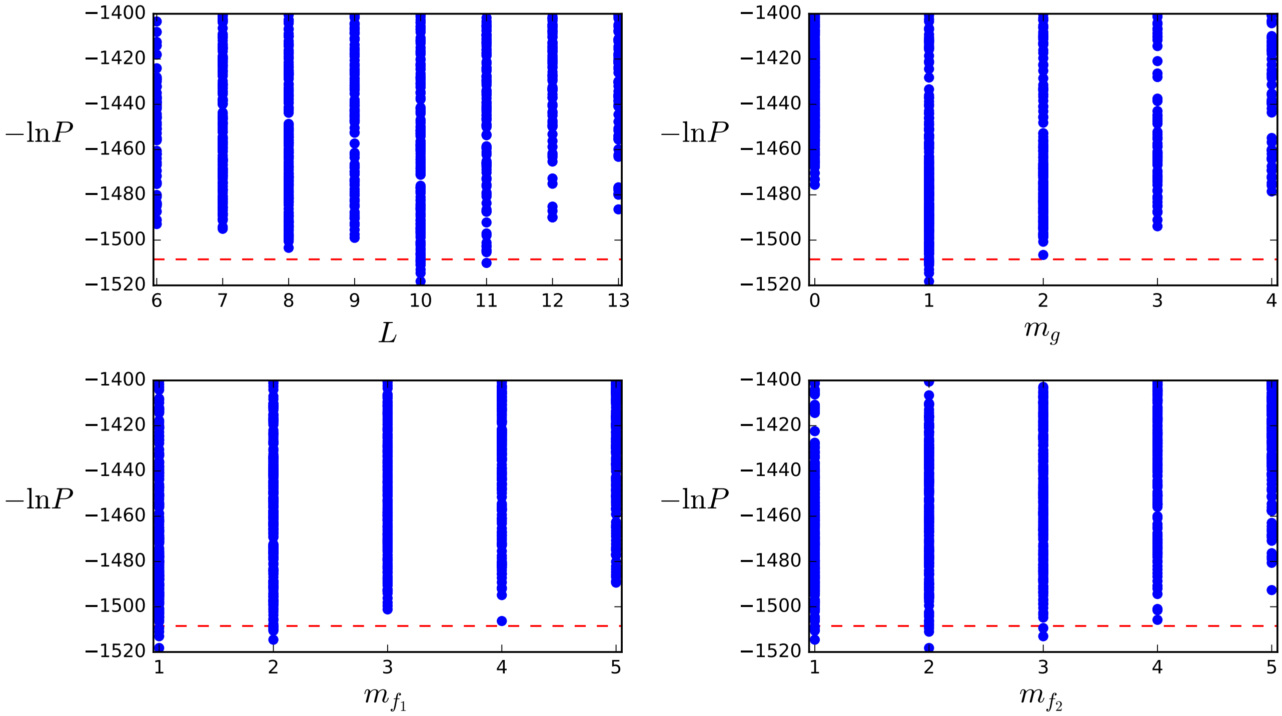

Figure S2. Model's optimality at different structural parameters. The dependences of the optimality Eq. 8 on the structural parameters L, m_g, m_{f_1} and m_{f_2} in the vicinity of the optimum are shown for the ensemble of learning iterations (the optimality is defined up to an arbitrary constant value). Dots below the red line correspond to the top-10 best models.

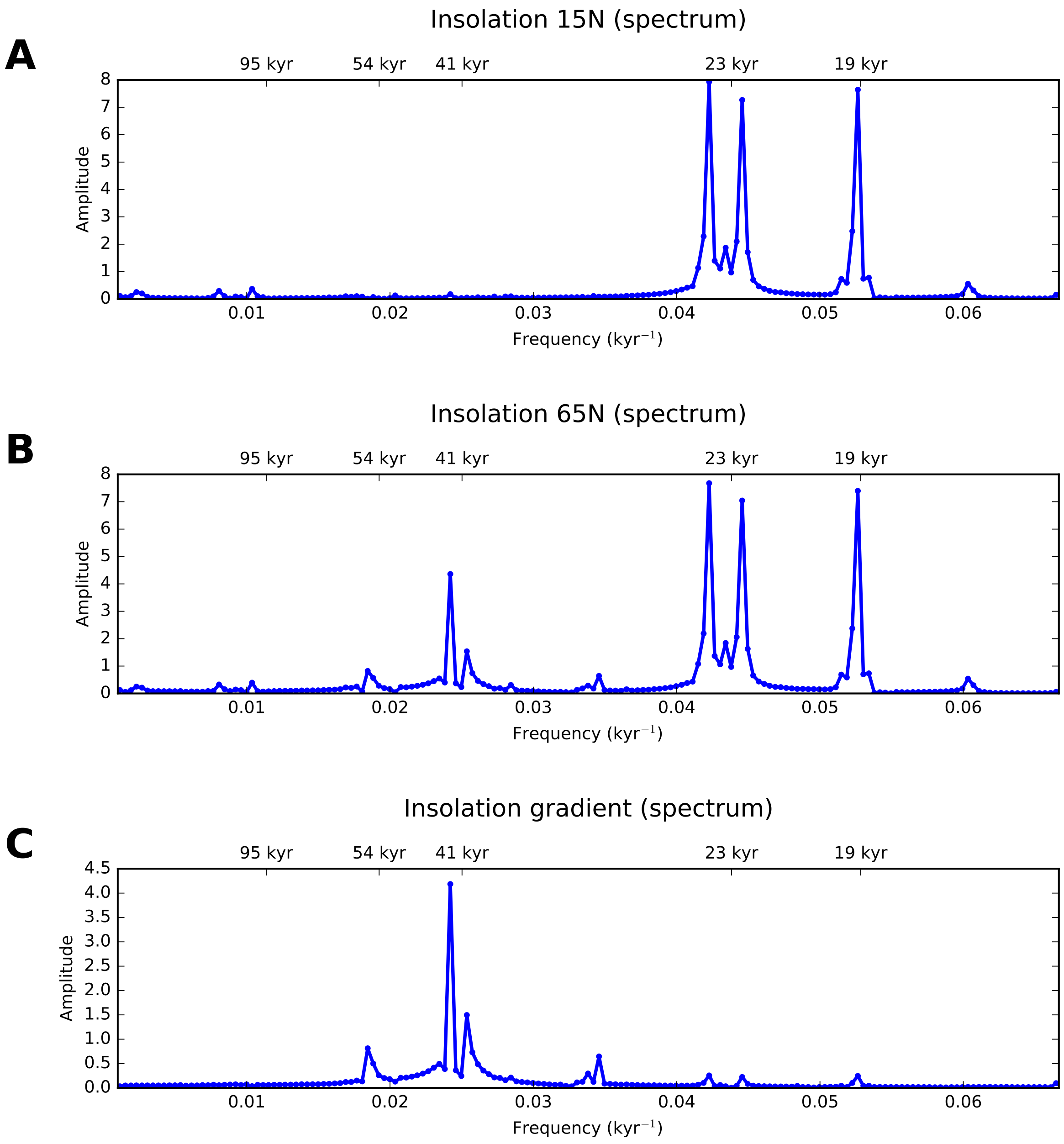

Figure S3. Fourier spectra of: (**A**) insolation at 15N latitude, (**B**) insolation at 65N latitude, (**C**) difference between them (the IG).

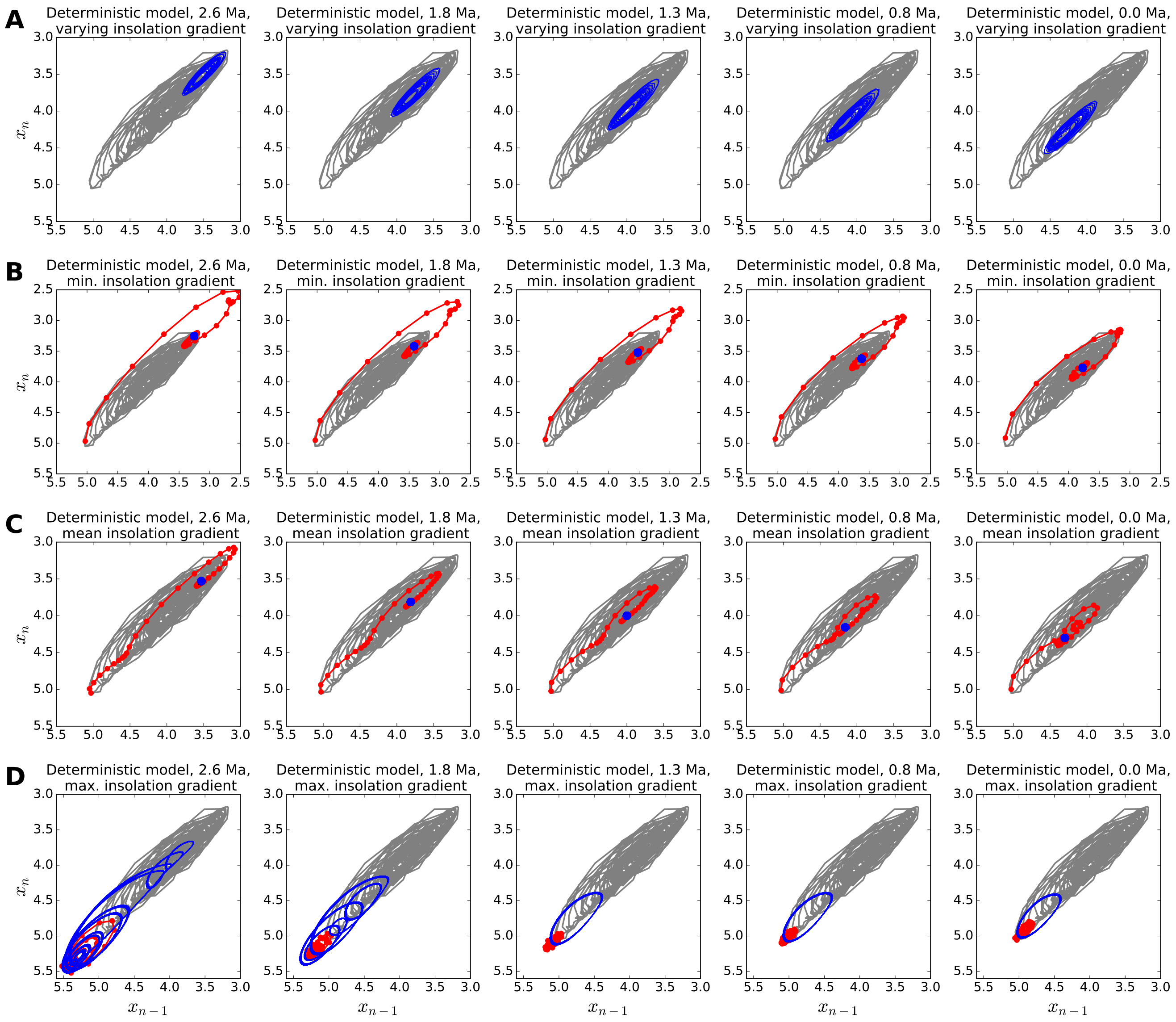

Figure S4. Two-dimensional (X_n, X_{n-1})-projection of the deterministic model's phase space: the best obtained model Eq. 1 with $g = 0$ and slow time t_n fixed at 2.6, 1.8, 1.3, 0.8 and 0 Ma (from left to right). On the top panels (**A**) blue lines show the 1 Myr-long trajectory of the deterministic system under the full time-varying forcing. On the other panels the behavior is shown for the model with insolation gradient fixed at its minimal (**B**), average (**C**) and maximal (**D**) values; here blue lines show the deterministic model's attractor, red lines – examples of the first 100 kyr of model's transient behavior from the "cold" initial conditions (high values of X). On all panels gray lines show the LR04 time series on the (X_n, X_{n-1}) plane as a background image. Examples of fast motions to the attractors from the cold states can be seen at the panels (B) and (C) ("red" trajectories).

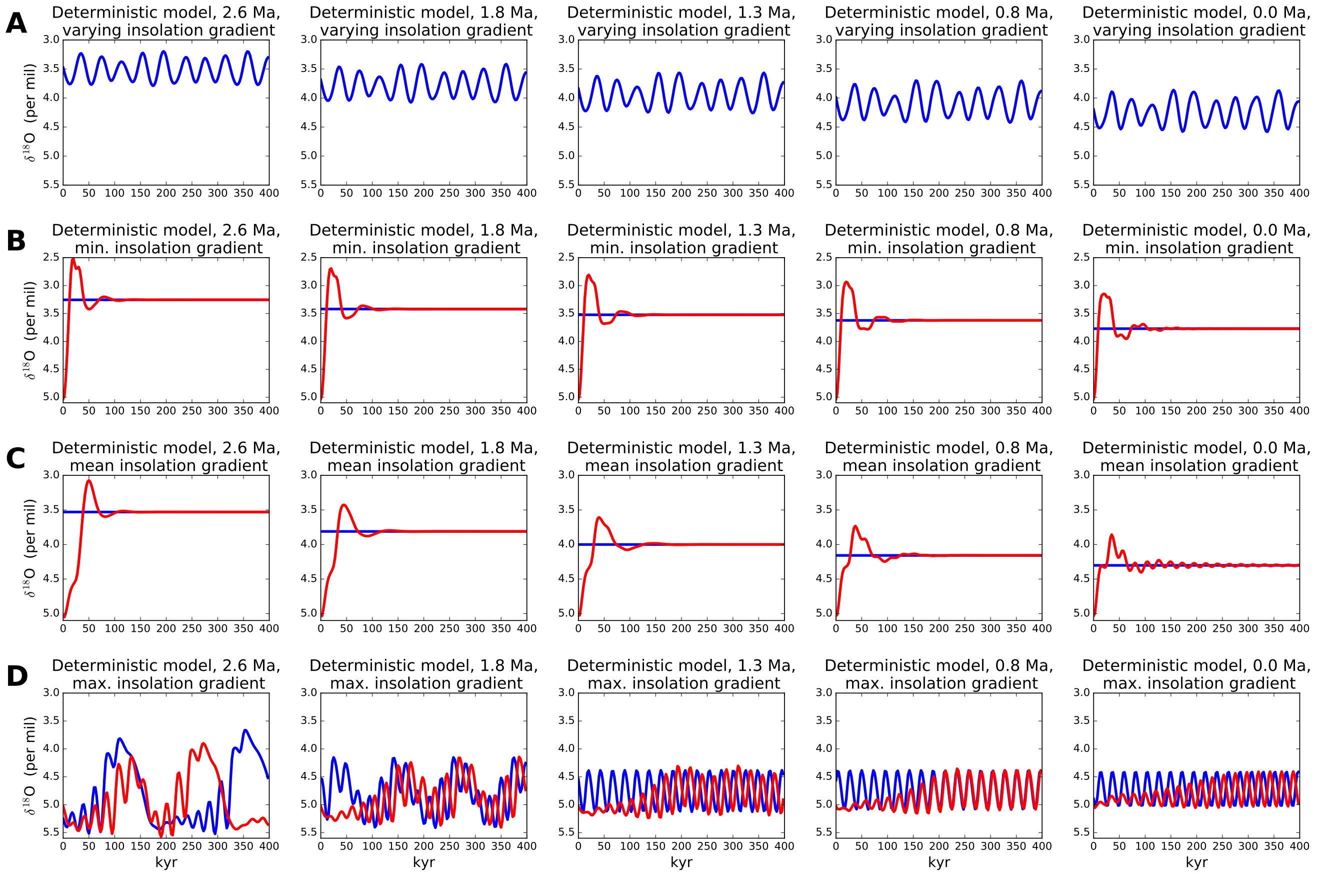

Figure S5. 400 kyr-long fragments of time series of X_n corresponding to the behavior shown in Fig. S4 by red and blue lines. In particular, "red" trajectories are longer than in Fig.S4 to demonstrate actual time of reaching the attractors.

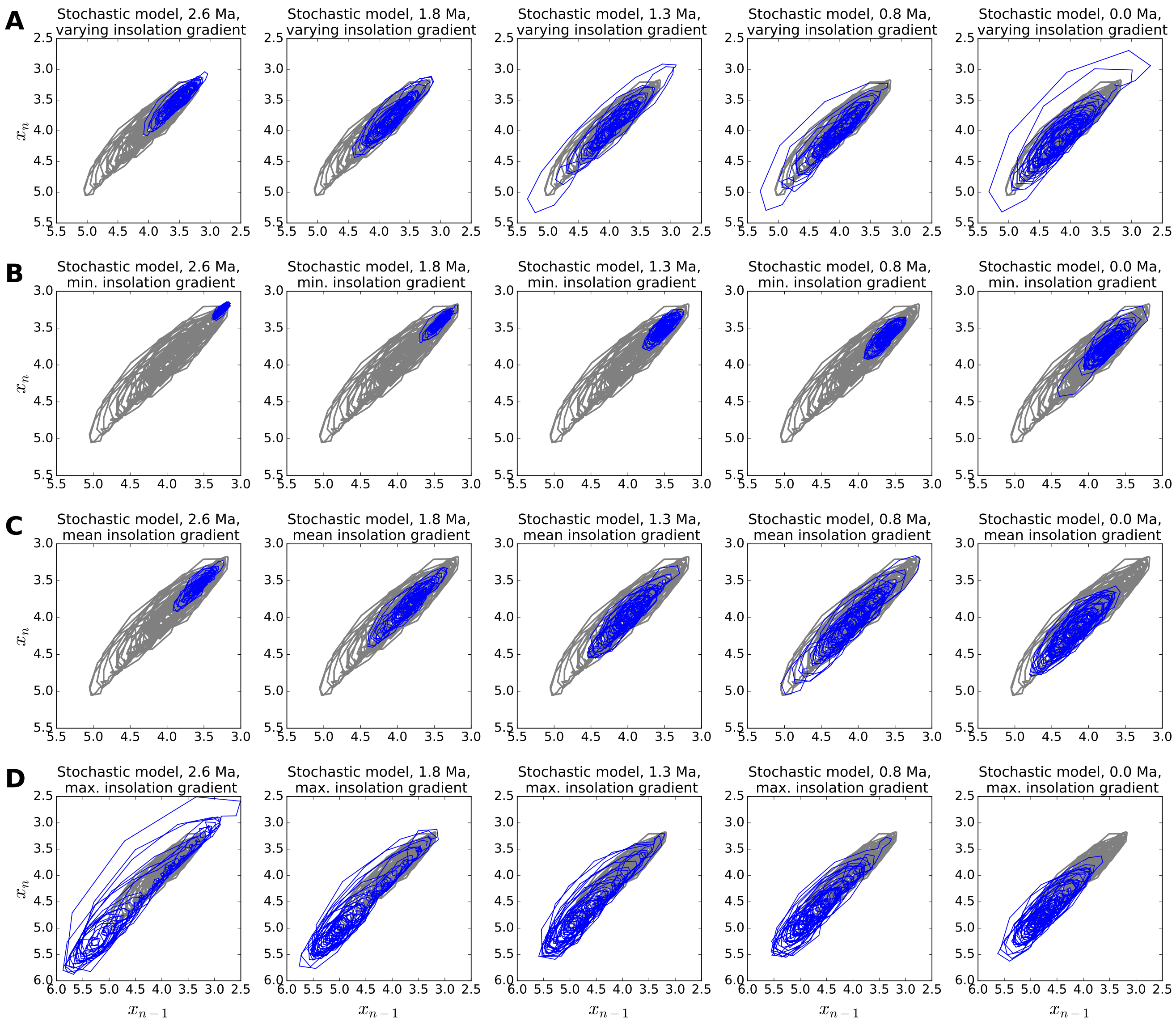

Figure S6. The behavior corresponding to the same cases as in Fig. S4 but for the full stochastic model Eq. 1 ($g \neq 0$). Blue lines show the randomly generated time series of 1.2 Myr-length on the (X_n, X_{n-1})-plane. Gray lines show the LR04 time series on the (X_n, X_{n-1}) plane as a background image.

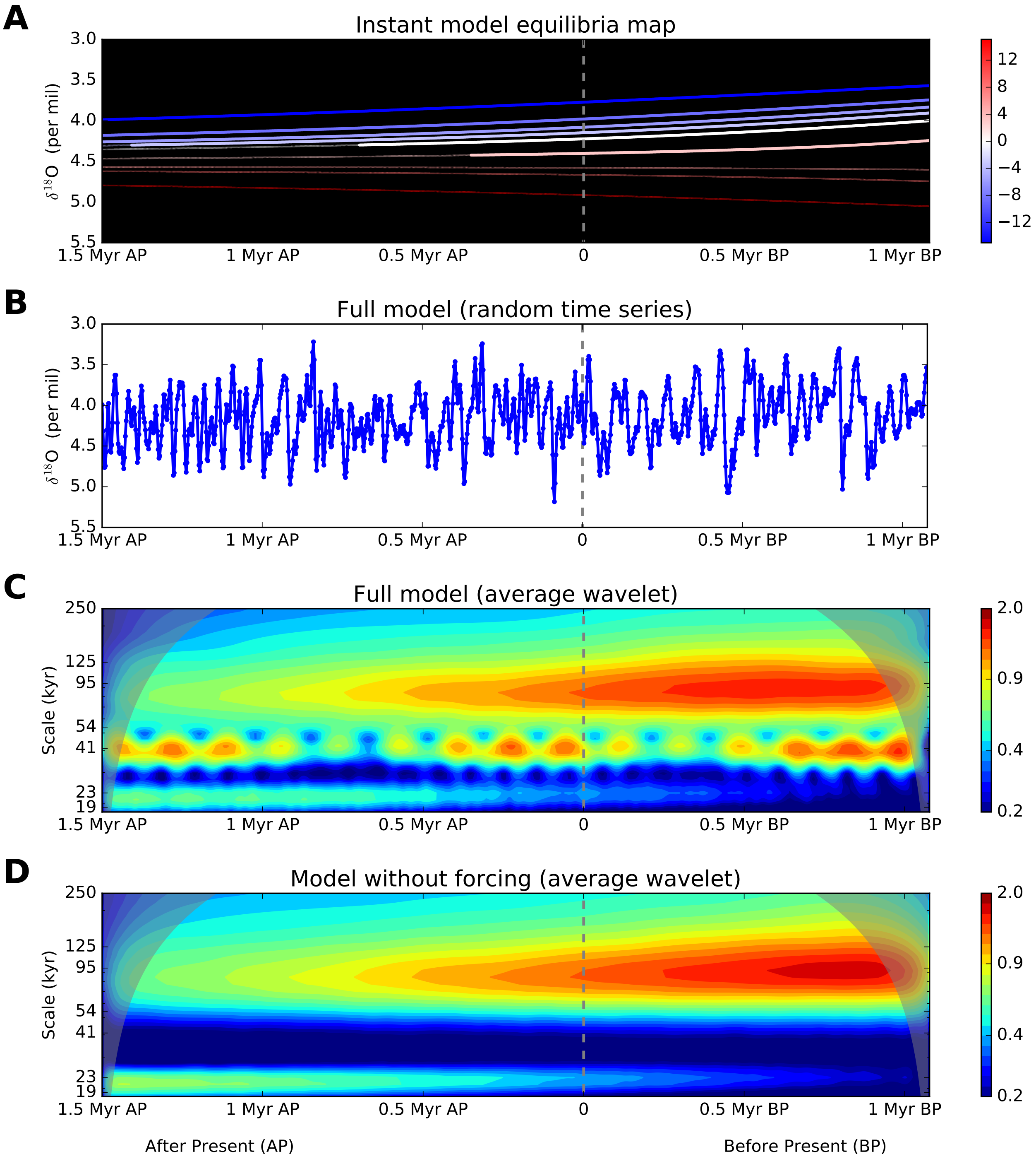

Figure S7. Behavior of the best obtained model extrapolated into the future. (**A**) The same as in Fig. 3 (B) but for time interval from 1 Myr before present (BP) to 1.5 Myr after present (AP). (**B**) Example of model time series on this time interval. (**C–D**) The same as in Figs. 1 (C–D) but for the interval from 1 Myr BP to 1.5 Myr AP.

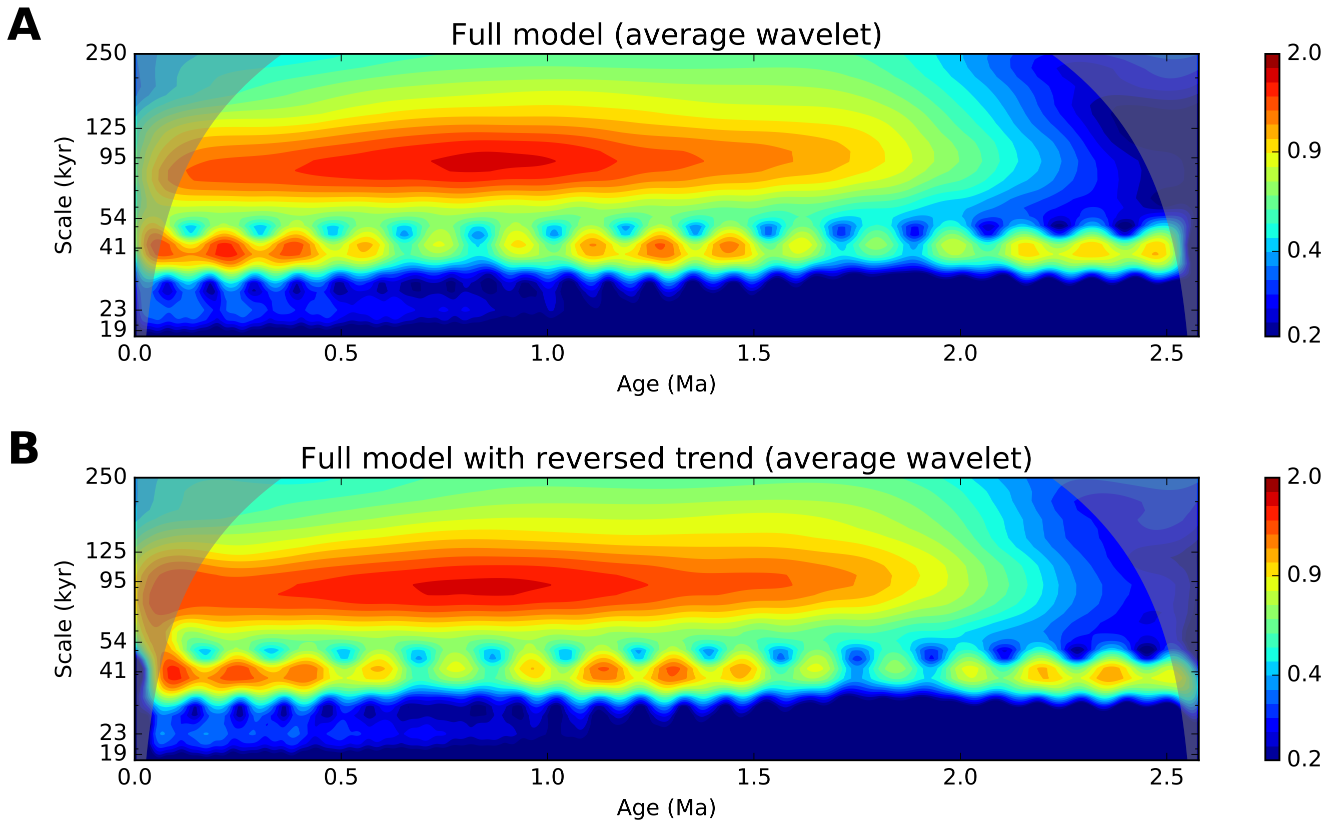

Figure S8. A comparison of the outputs of the models with direct (**A**) and reverse in time (**B**) trends (see the Discussion section in the main manuscript). The wavelet power spectra averaged over 10,000 model runs each, are shown.
